# Supplementary material for: Full-Length Transcriptome Profiling of Coridius chinensis Mitochondrial Genome Reveals the Transcription of Genes with Ancestral Arrangement in Insects
Source: Genes (Basel). 2023 Jan 15;14(1):225. doi: 10.3390/genes14010225 (PMC9859431; doi:10.3390/genes14010225)
Supplement: Supplementary file 1 [file genes-14-00225-s001.zip › genes-2121303-supplementary.pdf]

Supplementary Tables

Table S1. The mitochondrial polycistronic transcripts, antisense transcripts and lncRNAs from control region of *Coridius chinensis* mitochondrial genome.

Serial number	Polycistronic transcripts, antisense transcripts and lncRNAs from CR	Coding strand	position (J strand as reference)	Length/nt
71699058	trnM/ND2*	J	155-357	203
172952174	trnM/ND2*	J	155-357	203
108333789	trnM/ND2*	J	155-719	565
146670099	trnM/ND2*	J	155-773	619
168166122	trnM/ND2*	J	155-781	627
125173891	trnM/ND2	J	155-1204	1050
9830431	trnM/ND2*	J	156-406	251
67110025	trnM/ND2*	J	157-773	617
97190650	trnW/astrnC/astrnY/COX1*	J	1205-2179	975
17237992	*astrnC/astrnY/COX1/trnL/COX2	J	1269-3693	2425
11994350	*astrnC/astrnY/COX1*	J	1269-2179	911
30802161	*astrnC/astrnY/COX1*	J	1269-2179	911
62589691	*astrnC/astrnY/COX1*	J	1269-2179	911
108921764	*astrnC/astrnY/COX1*	J	1269-2179	911
111085959	*astrnC/astrnY/COX1*	J	1269-2179	911
164495598	*astrnC/astrnY/COX1*	J	1269-2434	1166
65143527	*astrnC/astrnY/COX1	J	1269-2945	1677
11993529	*astrnC/astrnY/COX1/trnL/COX2*	J	1269-3249	1981
17956950	*astrnC/astrnY/COX1*	J	1270-2179	910
108856142	*COX1/trnL/COX2	J	2243-3698	1456
121834097	*COX1/trnL/COX2	J	1693-3698	2006
163840600	*COX1/trnL/COX2	J	2369-3698	1330
21168277	*trnL/COX2*	J	2970-3225	256
85524510	trnL/COX2*	J	2949-3270	322
103416043	trnL/COX2*	J	2949-3395	447
42665688	trnL/COX2*	J	2949-3489	541
117967331	trnL/COX2*	J	2949-3687	739
153026922	trnL/COX2*	J	2949-3692	744
26214617	trnL/COX2	J	2949-3698	750
29361021	trnL/COX2	J	2949-3698	750
69994081	trnL/COX2	J	2949-3698	750
76416946	trnL/COX2	J	2949-3698	750
125766416	trnL/COX2	J	2949-3698	750
133368572	trnL/COX2	J	2949-3698	750
171510595	trnL/COX2	J	2949-3698	750
35915432	trnD/ATP8/ATP6	J	3775-4665	891
97714380	trnD/ATP8/ATP6	J	3775-4665	891
174393085	trnD/ATP8/ATP6	J	3775-4665	891
77007354	*trnD/ATP8/ATP6	J	3801-4665	865
151521295	*COX3/trnG/ND3	J	4913-5880	968
142213254	COX3/trnG/ND3/trnA	J	4666-5949	1284
114295198	trnG/ND3	J	5454-5880	427
163776606	trnG/ND3	J	5454-5880	427
44763965	trnG/ND3/trnA/trnR	J	5454-6017	564
72288869	trnA/trnR/trnN/trnS*	J	5881-6122	242
14942667	trnS/trnE/astrnF/asND5*	J	6085-7074	990
155714129	trnS/trnE/astrnF/asND5*	J	6085-7074	990
103483889	astrnF/asND5*	J	6219-6780	562
166265604	*asND5/astrnH/asND4*	J	6323-8449	2127
75104507	*asND5*	J	6891-7918	1028
12780348	*asND5*	J	6988-7937	950
83690892	*asND5*	J	7018-7571	554
140706493	*asND5/astrnH/asND4*	J	7136-8435	1300

80284123	*asND5/astrnH/asND4*	J	7375-8449	1075
116852750	*asND5/astrnH/asND4*	J	7437-8062	626
158859677	*asND5/astrnH/asND4*	J	7549-8449	901
46137738	*asND5/astrnH/asND4*	J	7858-8685	828
112199986	*asND4*	J	8109-8882	774
113904180	trnT/astrnP/ND6*	J	9668-10088	421
121045612	*trnT/astrnP/ND6	J	9686-10283	598
175112492	astrnP/ND6*	J	9733-10125	393
145689382	astrnP/ND6	J	9733-10283	551
3541110	astrnP/ND6/CYTB*	J	9733-10580	848
12845817	astrnP/ND6/CYTB*	J	9733-11403	1671
109969504	astrnP/ND6/CYTB	J	9733-11420	1688
169151163	astrnP/ND6/CYTB	J	9733-11420	1688
173474771	astrnP/ND6/CYTB	J	9733-11420	1688
22284284	ND6/CYTB*	J	9801-10677	877
5243673	*CR	J	15377-	566
95946640	*CR	J	15377-	857
119408095	*CR/trnI*	J	15378-45	857
127142199	*CR/trnI/astrnQ/trnM/ND2*	J	15379-408	1244
94570961	*astrnW/asND2/astrnM	N	1260-143	1118
160368949	*astrnW/asND2*	N	1260-279	982
93192919	*astrnW/asND2*	N	1223-428	796
98435186	*asCOX1	N	1743-1403	341
158795491	astrnD/astrnK/asCOX2/astrnL/asCOX1*	N	3842-2219	1624
151388464	*asCOX1*	N	2878-2504	375
20317147	*ND5/trnF/astrnE/astrnS/astrnN/astrnR/astrnA*	N	7609-5928	1682
151519544	trnH/ND5*	N	8058-7459	600
101714334	*asND6/trnP/astrnT/ND4L/ND4*	N	9889-8822	1068
127861783	*ND1/astrnS/asCYTB/asND6	N	11704-9798	1907
41290106	*asND6	N	10144-9798	347
14877222	*asCYTB/asND6	N	10573-9798	776
134348951	*asCYTB/asND6	N	10573-9798	776
58393631	*asCYTB/asND6	N	10634-9798	837
167773527	*asCYTB/asND6	N	10634-9798	837
172033889	*ND1/astrnS/asCYTB/asND6	N	11537-9798	1740
144704395	*ND1/astrnS/asCYTB/asND6	N	11749-9798	1952
147785853	*ND1/astrnS/asCYTB/asND6	N	11791-9798	1994
118751899	*ND1/astrnS/asCYTB/asND6*	N	11549-10189	1361
73925983	ND1/astrnS/asCYTB*	N	12433-10611	1823
173279751	ND1/astrnS/asCYTB*	N	12433-10727	1707
81529632	ND1/astrnS/asCYTB*	N	12433-10768	1666
151390825	*ND1/astrnS/asCYTB*	N	12099-11070	1030
53085058	*ND1/astrnS/asCYTB*	N	12279-11392	888
136907277	*trnL/ND1	N	12454-11508	947
46336280	*trnL/ND1	N	12454-11508	947
177867566	trnL/ND1	N	12500-11508	993
6097129	*lrRNA/trnL/ND1	N	12699-11508	1192
165939856	*lrRNA/trnL/ND1	N	12786-11508	1279
140050475	*lrRNA/trnL/ND1	N	12835-11508	1328
95226179	*lrRNA/trnL/ND1	N	12885-11508	1378
6752360	*lrRNA/trnL/ND1	N	13013-11508	1506
152176036	*lrRNA/trnL*	N	13687-12444	1244
167314352	*lrRNA/trnL*	N	13263-12448	816
176555798	*lrRNA/trnL*	N	12844-12455	390
93063166	*lrRNA/trnL*	N	13499-12455	1045
44958672	*lrRNA/trnL*	N	13273-12456	818
119406606	*lrRNA/trnL*	N	13687-12456	1232
115018213	*lrRNA/trnL*	N	13266-12458	809
167314195	*lrRNA/trnL*	N	13266-12469	798
11272833	*lrRNA/trnL*	N	13362-12469	894
48628105	*lrRNA/trnL*	N	13484-12469	1016
30803508	*lrRNA/trnL*	N	13484-12475	1010

19988571	*lrRNA/trnL*	N	12937-12478	460
9438918	*lrRNA/trnL*	N	13499-12478	1022
41290225	lrRNA/trnL*	N	13774-12478	1297
63899301	*lrRNA/trnL*	N	13687-12479	1209
108331376	*lrRNA/trnL*	N	13313-12480	834
162595865	*lrRNA/trnL*	N	13687-12481	1207
114099300	*lrRNA/trnL*	N	13128-12488	641
132907624	*lrRNA/trnL*	N	13263-12488	776
44827905	*lrRNA/trnL*	N	13265-12488	778
98370343	*lrRNA/trnL*	N	13265-12488	778
18090750	*lrRNA/trnL*	N	13362-12488	875
122224857	*lrRNA/trnL*	N	13430-12488	943
102434081	*lrRNA/trnL*	N	13498-12488	1011
147260003	lrRNA/trnL*	N	13774-12490	1285
162398372	*lrRNA/trnL*	N	12844-12490	355
143853399	*lrRNA/trnL*	N	13161-12490	672
91555321	*lrRNA/trnL*	N	13348-12490	859
146670351	*lrRNA/trnL*	N	13498-12490	1009
13500691	*lrRNA/trnL*	N	13498-12490	1009
37161214	*lrRNA/trnL*	N	13687-12490	1198
55248597	lrRNA/trnL*	N	13774-12490	1285
11405278	*lrRNA/trnL*	N	12844-12491	354
1508536	*lrRNA/trnL*	N	13362-12491	872
16973876	*lrRNA/trnL*	N	13362-12491	872
27724293	*lrRNA/trnL*	N	13362-12491	872
169739026	*lrRNA/trnL*	N	13495-12491	1005
63047974	*lrRNA/trnL*	N	13687-12491	1197
110036708	*lrRNA/trnL*	N	13687-12491	1197
84937484	*lrRNA/trnL*	N	13266-12492	775
163119291	*lrRNA/trnL*	N	13343-12492	852
175507228	*lrRNA/trnL*	N	13362-12492	871
132843169	*lrRNA/trnL*	N	13388-12492	897
41028435	*lrRNA/trnL*	N	13484-12492	993
149488191	*lrRNA/trnL*	N	13498-12492	1007
56756660	*lrRNA/trnL*	N	13687-12492	1196
114427475	*lrRNA/trnL*	N	13687-12492	1196
115411620	*lrRNA/trnL*	N	13687-12492	1196
175048307	*lrRNA/trnL*	N	13687-12492	1196
170132535	*lrRNA/trnL*	N	13347-12493	855
131532965	*lrRNA/trnL*	N	13161-12495	667
107022352	*lrRNA/trnL*	N	13362-12495	868
40503028	*lrRNA/trnL*	N	13484-12495	990
113901859	*lrRNA/trnL*	N	13687-12495	1193
170133548	*lrRNA/trnL*	N	13687-12495	1193
34605046	*lrRNA/trnL*	N	13484-12496	989
19529819	srRNA/trnV/lrRNA*	N	14660-13750	911
41746703	srRNA/trnV*	N	14662-13811	852
74056825	srRNA/trnV*	N	14661-13813	849
150077470	srRNA/trnV*	N	14661-13820	842
154731009	srRNA/trnV*	N	14661-13824	838
113378294	*CR/srRNA	N	14841-13845	997
138412294	*CR/srRNA	N	15189-12846	2344
167969819	*CR/srRNA*	N	14713-13850	864
113117329	*CR/srRNA*	N	14710-13938	773
30934540	*CR*	N	14972-14901	72
9307853	*CR*	N	15027-14901	127
149685560	*CR*	N	15027-14901	127
134283344	*CR*	N	15028-14901	128
97583892	*CR*	N	15189-14901	289
90702191	astrnI/CR*	N	72-15406	862

* represents the incomplete transcripts.

Table S2. The mitochondrial tRNAs of *Coridius chinensis*.

Serial number	tRNA	position of tRNA gene	Coding strand	position of tRNA (J strand as reference)	Length/nt
169739742	*trnI	1-71	J	19-73	55
57214011	trnD	3775-3842	J	3775-3844	70
50266369	*trnG	5454-5518	J	5463-5520	58
108265960	trnG*	5454-5518	J	5454-5503	50
126943323	trnG	5454-5518	J	5454-5520	67
135005976	trnG	5454-5518	J	5454-5520	67
113969861	trnA	5881-5949	J	5881-5951	71
118950572	trnA	5881-5949	J	5881-5951	71
133237599	trnA	5881-5949	J	5881-5951	71
21956166	trnA	5881-5949	J	5881-5951	71
120718776	trnA	5881-5949	J	5881-5950	70
47711722	trnA	5881-5949	J	5881-5951	71
23530247	*trnN	6019-6084	J	6035-6086	52
52758504	*trnN	6019-6084	J	6035-6086	52
60558065	*trnN	6019-6084	J	6035-6086	52
90112428	*trnN	6019-6084	J	6035-6086	52
98239177	trnS	6085-6153	J	6085-6154	70
164364335	trnS	6085-6153	J	6085-6155	71
166922049	trnS	6085-6153	J	6085-6155	71
149618927	*trnS	6085-6153	J	6092-6154	63
82444884	*trnT	9668-9732	J	9685-9734	50
24642121	trnH	8057-7992	N	8058-7989	70
35389662	trnH	8057-7992	N	8058-7990	69
158206266	trnV	13841-13775	N	13841-13773	69
21629291	*trnV	13841-13775	N	13827-13774	54
175639039	*trnV	13841-13775	N	13833-13774	60

* represents the incomplete transcripts.
